# A mathematical model of bone remodeling dynamics for normal bone cell populations and myeloma bone disease

**DOI:** 10.1186/1745-6150-5-28

**Published:** 2010-04-20

**Authors:** Bruce P Ayati, Claire M Edwards, Glenn F Webb, John P Wikswo

**Affiliations:** 1 Department of Mathematics, University of Iowa, Iowa City, IA 52242, USA; 2 Vanderbilt Center for Bone Biology, Department of Cancer Biology, Vanderbilt University Medical Center, Nashville, TN 37232, USA; 3 Department of Mathematics, Vanderbilt University, Nashville, TN, 37235, USA; 4 Departments of Biomedical Engineering, Molecular Physiology and Biophysics, and Physics and Astronomy, Vanderbilt University, Nashville, TN 37235, USA; 5 Vanderbilt Institute for Integrative Biosystems Research and Education, 6809 Stevenson Center, VU Station B 351807 | Nashville, TN 37235-180

## Abstract

**Background:**

Multiple myeloma is a hematologic malignancy associated with the development of a destructive osteolytic bone disease.

**Results:**

Mathematical models are developed for normal bone remodeling and for the dysregulated bone remodeling that occurs in myeloma bone disease. The models examine the critical signaling between osteoclasts (bone resorption) and osteoblasts (bone formation). The interactions of osteoclasts and osteoblasts are modeled as a system of differential equations for these cell populations, which exhibit stable oscillations in the normal case and unstable oscillations in the myeloma case. In the case of untreated myeloma, osteoclasts increase and osteoblasts decrease, with net bone loss as the tumor grows. The therapeutic effects of targeting both myeloma cells and cells of the bone marrow microenvironment on these dynamics are examined.

**Conclusions:**

The current model accurately reflects myeloma bone disease and illustrates how treatment approaches may be investigated using such computational approaches.

**Reviewers:**

This article was reviewed by Ariosto Silva and Mark P. Little.

## Background

Bone is continually renewed throughout the skeleton in a process known as remodeling. The bone remodeling process is spatially heterogeneous, with regular but asynchronous cycles at multiple sites that can occupy 5-25% of bone surface [1]. In this way every part of the skeleton is remodeled periodically over time. Mathematical modeling of bone remodeling has focused on various aspects of this process. These approaches include models of how Michaelis-Menten-like feedback mechanics affect bone resorption (the process by which osteoclasts break down bone, resulting in bone loss) [2] and how biomechanical stress induces bone formation [3-6]. Other modeling efforts have examined the signaling pathways between osteoclasts and osteoblasts involved in bone remodeling [7], or accounted for the activity of both osteoclasts and osteoblasts in a microenvironment known as a basic multicellular unit (BMU) [8-12].

In this paper we develop mathematical models of myeloma bone disease. Multiple myeloma is a hematological malignancy associated with clonal expansion of malignant plasma cells within the bone marrow. One of the major clinical features of myeloma is the development of a progressive and destructive osteolytic bone disease, associated with severe bone pain, pathological fractures, osteoporosis, hypercalcemia and spinal cord compression. Interactions between myeloma cells and cells of the bone marrow microenvironment are critical for myeloma growth and survival and for the development of the osteolytic bone disease [13,14]. The destructive nature of myeloma bone disease is increased by the vicious cycle that develops between myeloma cells and the bone marrow microenvironment. Although the precise molecular mechanisms responsible for the bone destruction in multiple myeloma are not completely understood, it is known that the bone destruction is primarily mediated by osteoclasts, and that this destruction is exacerbated by a reduction in osteoblastic bone formation. Patients with multiple myeloma have abnormal bone remodeling, where resorption and formation become uncoupled, with the end result being an increase in bone resorption and a decrease in bone formation. Myeloma cells are found in close association with sites of active bone resorption, and their ability to stimulate osteoclast formation and activity has been well characterized [14-17]. Histological studies have demonstrated that in the early stages of myeloma, bone formation is actually increased, and this is thought to reflect the attempt to compensate for the increase in osteoclastic resorption [15]. However, as the disease progresses, bone formation is rapidly decreased [15,18,19]. This has been confirmed in studies which demonstrate that markers of bone formation are decreased in patients with multiple myeloma [20,21]. Despite many significant advances in the understanding of the biology of multiple myeloma, it remains an incurable malignancy, and the destructive osteolytic bone disease is a major cause of morbidity in patients with multiple myeloma.

## Results

**Figure 1:**
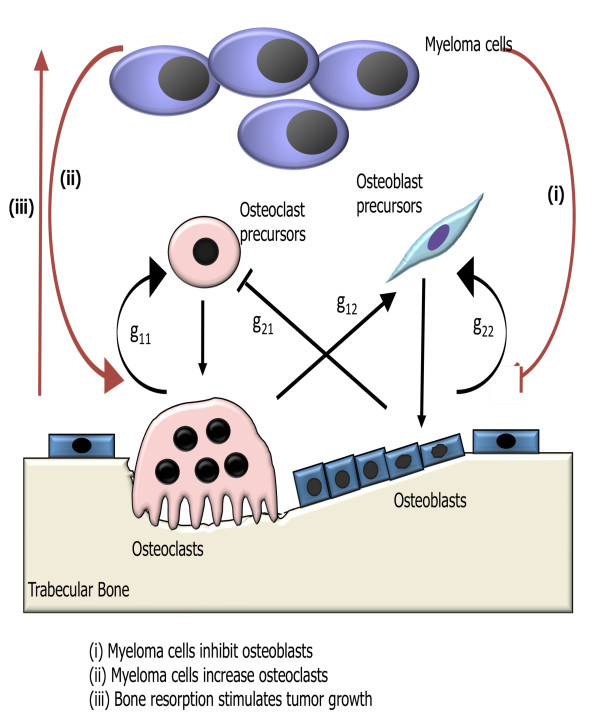
**Schematic of the effects of myeloma on the autocrine and paracrine signaling in the osteoclast and osteoblast cell populations in the presence of tumor**. The model of bone remodeling without tumor is taken from [22], including the meaning of the parameters *g*_11 _(osteoclast autocrine signaling), *g*_12 _(osteoclast stimulation of osteoblast production), *g*_21 _(osteoblast inhibition of osteoclast production), and *g*_22 _(osteoblast autocrine signaling). The tumor cells alter these interactions through modifications of the parameters *g*_11_, *g*_12_, *g*_21_, *g*_22_.

We model the influence of tumor growth on bone remodeling, and in particular how the tumor influences autocrine and paracrine signaling in the osteoclast and osteoblast cell populations (see Fig. 1). Autocrine signaling represents the feedback from osteoclasts and osteoblasts to regulate their respective formation. Paracrine signaling represents the factors produced by osteoclasts that regulate osteoblast formation, and vice versa. We use the underlying model of bone remodeling in the absence of tumor presented in [22] and explored further in [23-25]. This model is a dynamical system with zero explicit space dimensions, but with a dependent variable that records bone mass as a function of time. If we interpret the bone mass equation as one for localized trabecular mass (spongy bone found within the bone marrow) underneath a point on the surface of the bone, we obtain a representation of one spatial dimension. We then present one-dimensional spatial models that suggest how we may incorporate additional spatial dimensions.

The organization of the paper is as follows: In Section 2 we first present the tumor-free zero-dimensional model in [22] and illustrate its simulation of normal bone modeling dynamics. In Section 3 we add the tumor cell population to the model of normal bone in Section 2, and in Section 4 we simulate treatment for the model in Section 3. In Section 5 we add an explicit spatial dimension to the normal bone model. This additional dimension allows for heterogeneity on the surface of the bone along one axis. In Sections 6 and 7 we add tumor and treatment to the spatial model. In Section 8 we conclude with a discussion of the results. Computations were conducted using the *Mathematica *function NDSolver. All models in this paper use dimensionless variables and parameters, including for the cell populations.

### Zero-dimensional Bone Model without Tumor

A model of normal bone remodeling at a single discrete site is developed in [22]. The model consists of a system of ordinary differential equations describing the bone cell populations in a BMU. These populations are the osteoclasts, which resorb bone, and osteoblasts, which form bone. The variables of the model are the density of osteoclasts *C*(*t*) and the density of osteoblasts *B*(*t*) at time *t*. The equations of the model in [22] are(1)

with initial conditions *C*(0) = *C*_0_, *B*(0) = *B*_0_. The power law nonlinearities in (1a) and (1b) are approximations for the interactions of the osteoclast and osteoblast populations in the proliferation terms of the equations. In (1a) autocrine signaling has a positive feedback on osteoclast production (*g*_11 _> 0), and paracrine signaling has a negative feedback on osteoclast production (*g*_21 _< 0). In (1b) autocrine signaling has a positive feedback on osteoblast production (*g*_22 _> 0), and paracrine signaling has a positive feedback on osteoblast production (*g*_12 _> 0).

The system (1) has a unique nontrivial steady state(3)

where(5)

The system (1) has periodic solutions when(6)

The solutions exhibit limit cycles as Ψ passes through 0: Ψ < 0 yields damped oscillations converging to the nontrivial steady state , , and Ψ > 0 yields unstable oscillations diverging away from the nontrivial steady state (where Ψ is sufficiently close to 0).

An additional variable *z*(*t*) for the bone mass is obtained in [22] by assuming bone mass is determined by the extent to which normalized values of *C*(*t*) and *B*(*t*) exceed nontrivial steady state levels. We reinterpret the variable *z*(*t*) as representing localized trabecular mass beneath a point on the bone surface. Having found the solutions of system (1), we define the following equation for the bone mass *z*(*t*) at time *t*:(7)

with the initial condition *z*(0) = *z*_0_. In the case that *C*(*t*) and *B*(*t*) have periodic solutions, *k*_1 _and *k*_2 _are chosen so that the normal bone mass oscillates with the same periodicity as the osteoclast and osteoblast populations with an experimentally determined amplitude of the oscillations. The assumption is that bone mass decreases or increases cyclically according to the net effects of resorption (*C*(*t*) >) and formation (*B*(*t*) >). The constants *k*_1 _and *k*_2 _satisfy *k*_1 _= *rR*, and *k*_2 _= *r*, where(8)

 is the period of the cycles of *C*(*t*) and *B*(*t*), and *r *is determined by the amplitude of the oscillations in the bone mass. The value *R *is well-defined as long as *B*(0) ≠ .

**Figure 2:**
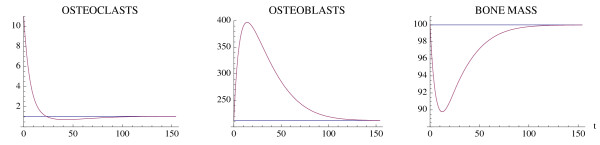
**A single event of normal bone remodeling initiated by a momentary perturbation of the osteoclast nontrivial steady state population by an increase of 10 cell units**. System of equations (1): The osteoclast population first decreases with a consequent increase in osteoblast population and a resorption of bone mass, followed by a return to steady-state levels. The blue line represents the steady-state solution. The parameters are *α*_1 _= 3.0, *α*_2 _= 4.0, *β*_1 _= 0.2, *β*_2 _= .02, *g*_11 _= .5, *g*_22 _= 0.0, *g*_12 _= 1.0, and *g*_21 _= -0.5. The nontrivial steady state with these parameters is  = 1.06 and  = 212.13. The initial conditions are *C*(0) = 11.06 and *B*(0) = 212.13. The bone mass parameters are *k*_1 _= .24, *k*_2 _= .0017, as in [22].

**Figure 3:**
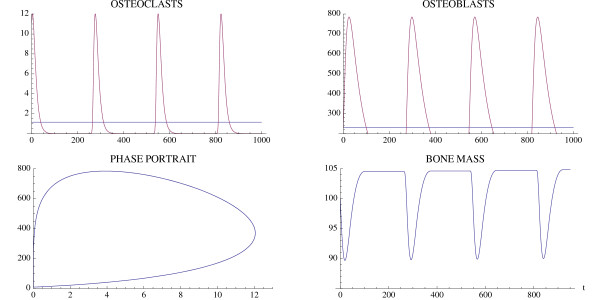
**Simulation of oscillatory changes in osteoclast and osteoblast populations during normal bone modeling for which the model solutions are periodic**. System of equations (1): The oscillations are stimulated by an initial increase in the number of osteoclasts by 10 cell units above the nontrivial steady state. The parameters are the same as in Fig. 2, except that *g*_11 _= 1.1. The nontrivial steady state is  = 1.16,  = 231.72, the initial conditions are *C*(0) = 11.16, *B*(0) = 231.72, and the bone mass parameters are *k*_1 _= .0748, *k*_2 _= .0006395. The bone mass oscillates about a normalized value of 100.

We reproduce here two examples of single-site normal bone remodeling given in [22]. The parameters are as in [22], with time unit in days. Fig. 2 simulates a targeted event corresponding to an external stimulus, taken as a perturbation of the nontrivial steady state (, ) by a momentary increase in the number of osteoclasts. The result is a single remodeling cycle (Ψ < 0, but not sufficiently close to 0 to yield damped oscillations). Fig. 3 corresponds to a series of internally initiated regular cycles over an extended period of time, with osteoclast and osteoblast populations exhibiting regular periodic cycles about their nontrivial steady state values (Ψ = 0). The distinction of the two behaviors is the value of the osteoclast autocrine parameter *g*_11_, which is greater in Fig. 3. In [22] it is claimed that this parameter is the primary factor in the regulation of bone remodeling dynamics.

### Zero-dimensional Bone Model with Tumor

We find that the regular cycles of the normal bone model above are perturbed by the presence of myeloma. The tumor parameters yield perturbations of the normal cycles that result in limit cycles, which are either damped oscillations that converge to the nontrivial steady state or undamped oscillations that diverge away from the nontrivial steady state. The variables for the model with tumor are *C*(*t*), *B*(*t*) as before, and *T*(*t*) = density of tumor cells at time *t*. Our equations are(9)

with the initial conditions *C*(0) = *C*_0_, *B*(0) = *B*_0_, and *T *(0) = *T*_0_. The equation for bone mass *z*(*t*) is (5), as before. The tumor equation (7c) is of Gompertz form with growth constant γ_*T*_> 0 and maximum tumor size *L*_*T*_. We have taken γ_*T *_to be independent of bone loss. Future work will include models, simulations, and biological experiments to determine the dependence of γ_*T *_on bone loss. In (7) the tumor parameters *r*_11_, *r*_12_, *r*_21_, *r*_22 _are all nonnegative.

The tumor presence alters (1a) as follows: autocrine promotion of osteoclasts is increased (, since *g*_11 _> 0); paracrine inhibition of osteoclasts is reduced (, since *g*_21 _< 0); paracrine promotion of osteoblasts is reduced (, since *g*_12 _> 0); and autocrine promotion of osteoblasts is reduced (, since *g*_22 _> 0). The key difference between our model and that of [22] in (1) is the addition of the terms *r*_*ij*_*T *(*t*)/*L*_*T *_that couple the tumor density and maximum size to the power laws for the osteoclast/osteoblast interactions.

The system (7) has nontrivial steady states(12)

and(13)

Where(16)

The system (7) has stable cycles when(17)

If Φ < 0, then the system exhibits damped oscillations converging to the nontrivial steady state, and if Φ > 0, then the system exhibits unstable oscillations converging away from the nontrivial steady state (where Φ is sufficiently close to 0).

**Figure 4:**
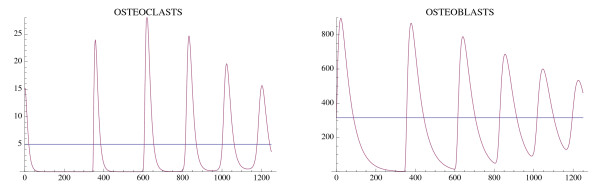
**The osteoclast and osteoblast populations in the presence of tumor stimulated by an initial osteoclast population elevated above the nontrivial steady state by 10 cell units**. System of equations (7): The osteoclast population first increases as the osteoblast population decreases. The solutions converge to the nontrivial steady state  = 5.0 and  = 316.0 with damped oscillations. The parameters are *α*_1 _= 3.0, *α*_2 _= 4.0, *β*_1 _= 0.2, *β*_2 _= .02, *g*_11 _= 1.1, *g*_22 _= 0.0, *g*_12 _= 1.0, *g*_21 _= -0.5, γ_*T *_= .005, *L*_*T *_= 100, *r*_11 _= .005, *r*_21 _= 0.0, *r*_12 _= 0.0, *r*_22 _= 0.2. The initial conditions are *C*(0) = 15.0, *B*(0) = 316.0, *T*(0) = 1.

**Figure 5:**
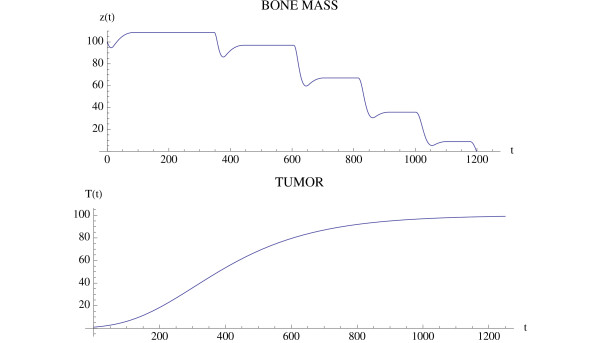
**Bone mass and tumor response to the oscillations in Fig. 4**. System of equations (7): The bone mass converges with oscillations to 0.0 and the tumor converges to maximum capacity *L*_*T*_. The parameters are as in Fig. 4. The bone mass parameters are *k*_1 _= .0748, *k*_2 _= .0006395 as in Fig. 3.

A simulation of the bone model with tumor is given in Fig. 4 and Fig. 5 with Φ < 0. The osteoclast and osteoblast populations exhibit damped oscillations converging to the nontrivial steady state in Fig. 4, the tumor grows to maximum capacity, and the bone mass converges with oscillations to 0 (Fig. 5). We can see that as the tumor burden increases, there is an initial increase in both osteoclast and osteoblast number, reflecting the attempt of the system to maintain the normal coupling of bone resorption to bone formation even in the presence of tumor. Although the amplitude of osteoclast oscillations decreases over time, this reflects the decrease in bone mass. Importantly, the amplitude of osteoclast oscillations in the presence of tumor is higher than when tumor cells are not present (e.g., Fig. 3), reflecting the overall increase in osteoclasts associated with myeloma bone disease. In contrast, the amplitude of osteoblast oscillations is dramatically decreased when compared with the non-tumor simulation (Fig. 3), indicating the decrease in osteoblasts which is a characteristic feature of myeloma bone disease.

**Figure 6:**
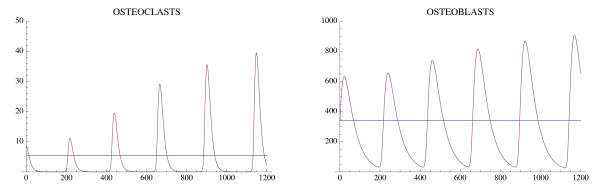
**The osteoclast and osteoblast populations in the presence of tumor exhibit unstable oscillations**. System of equations (7): The parameters are as in Fig. 4 except *r*_11 _= .02. The nontrivial steady state is  = 5.46 and  = 340.52. The initial conditions are *C*(0) = 8.46, *B*(0) = 340.52, *T*(0) = 1.

**Figure 7:**
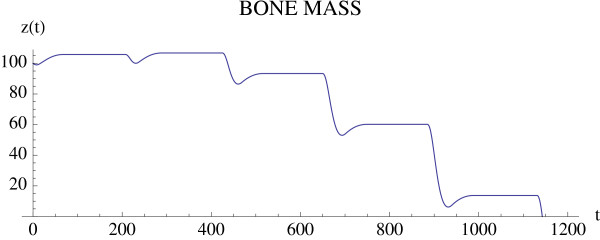
**The effect of the unstable oscillations in Fig. 6 on bone mass**. System of equations (7): The bone mass decreases with oscillations to 0.0. The tumor converges to maximum capacity *L*_*T *_as in Fig. 5. The parameters are as in Fig. 4, except that *r*_11 _= .02. The bone mass parameters are *k*_1 _= .0748, *k*_2 _= 0006395 as in Fig. 3.

Another simulation of the bone model with tumor is given in Fig. 6 and Fig. 7 with the only change from Fig. 4 and Fig. 5 being that *r*_11 _= .02 instead of .005. In this case the osteoclast and osteoblast populations exhibit unstable oscillations (Φ > 0), whose amplitude grows with time, with a concomitant decrease in bone mass and increased growth of the tumor to its limiting size (Fig. 7).

**Figure 8:**
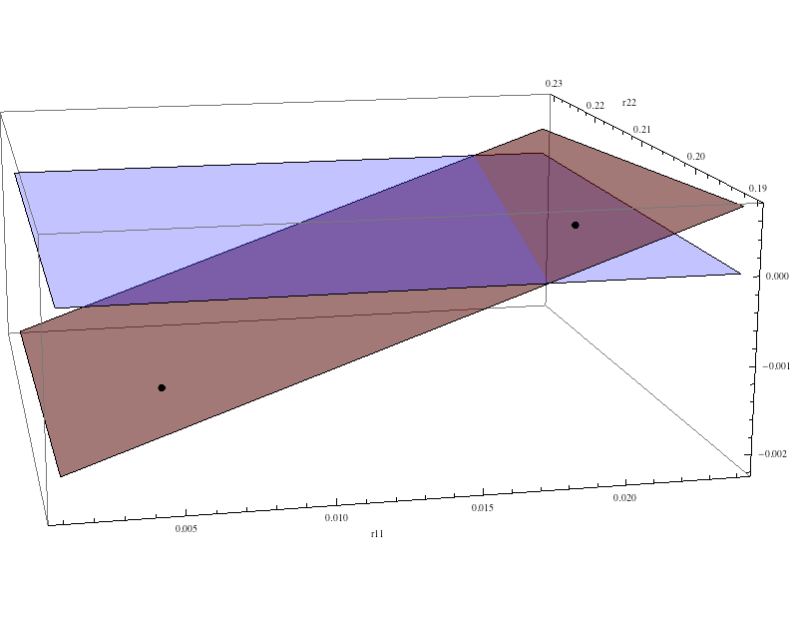
**The behavior of the solutions as a function of the tumor parameters *r*_11 _and *r*_22_**. System of equations (7): The red surface is the plot of Φ = *β*_1_(*g*_11_(1 + *r*_11_) 1) + *β*_2_(*g*_22 _- *r*_22 _- 1) as a function of *r*_11 _and *r*_22 _(as in (11)). If the point on the surface corresponding to (*r*_11_, *r*_22_) is negative, then the solutions have decreasing amplitude oscillations converging to the nontrivial steady state; if positive, then the solutions have increasing amplitude and unstable oscillations. The values *r*_11 _= .005 and *r*_22 _= 0.2 in Fig. 4 and Fig. 5 correspond to -.00145 on the red surface, and the solutions converge slowly to the nontrivial steady state  = 5.0,  = 316.0. The values *r*_11 _= .02 and *r*_22 _= 0.2 in Fig. 6 and Fig. 7 correspond to .0002 on the red surface, and the solutions are unstable. The other parameters are *r*_12 _= 0, *r*_21 _= 0, *α*_1 _= 3.0, *α*_2 _= 4.0, *β*_1 _= 0.2, *β*_2 _= .02, *g*_11 _= 1.1, *g*_22 _= 0.0, *g*_12 _= 1.0, *g*_21 _= -0.5, γ_*T *_= .005, *L*_*T *_= 100.

As in [22] we can analyze the behavior of the model in terms of selected parameters. Here we investigate the dependence of the solution behavior on the parameters *r*_11 _and *r*_22 _and fix all the other parameters. The model has a unique nontrivial steady state as in (9a),(9b),(9c), which depends on *r*_11 _and *r*_22_. The behavior of the solutions of the model as a function of *r*_11 _and *r*_22 _depends on the eigenvalues of the Jacobian at the nontrivial steady states (, ). If the maximum of the real parts of the eigenvalues is less than 0, then the solutions converge to the nontrivial steady state with damped oscillations, and if the maximum is greater than 0, then the solutions have unstable oscillations. These cases are illustrated in Fig. 8 as a function of *r*_11 _and *r*_22_. The parameters *r*_11 _and *r*_22 _correspond to the alteration of the normal osteoclast-osteoblast regulation due to tumor burden. The relative values of *r*_11 _and *r*_22 _yield the two types of instability, that is, either decreasing amplitude oscillations or increasing amplitude oscillations, both departing from normal stable periodic oscillations.

### Zero-dimensional Bone Model with Tumor and Drug Treatment

Many therapeutic approaches for the treatment of myeloma have the potential to affect directly both myeloma cells and cells of the bone marrow microenvironment, including osteoclasts and osteoblasts. Therefore, it is difficult to predict from *in vivo *studies the overall response to drug treatment in myeloma. We present a framework which provides us with the opportunity to model, for the first time, the effects of drug treatment on oscillatory bone remodeling. We have chosen to model the effects of proteasome inhibition in myeloma bone disease. Proteasome inhibitors are known to have direct anti-myeloma effects, and to have direct effects on osteoblasts to stimulate osteoblast differentiation and bone formation [26-30]. The equations are as before except that the treatment promotes osteoblast production and inhibits tumor growth:(18)

**Figure 9:**
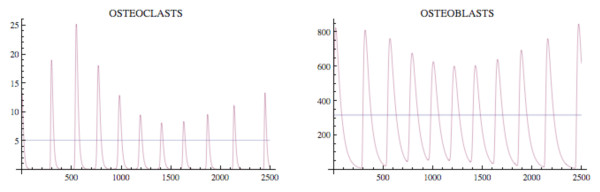
**Treatment of the tumor model in Fig. 4 starts at *t*_*start *_= 600 with intensity values *v*_1 _= .001 and *v*_2 _= .008**. System of equations (7): Treatment reverses the disruption of the osteoclasts' and osteoblasts' interaction induced by the tumor (compare to Fig. 4). The parameter values are as in Fig. 4 and the initial conditions are *C*(0) = 13.0 and *B*(0) = 300.0.

**Figure 10:**
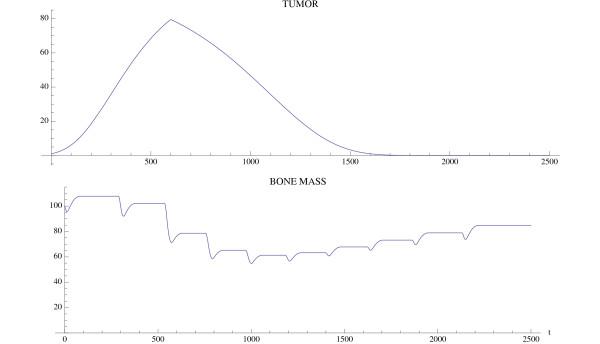
**The tumor is extinguished and the bone mass begins to recover (compare to Fig. 5)**. System of equations (7): The parameters are as in Fig. 9 and the bone mass parameters are *k*_1 _= .0748, *k*_2 _= 0006395 as in Fig. 3.

The time-dependent treatment functions *V*_1_(*t*) and *V*_2_(*t*) in (12b) and (12c) are(21)

where *t*_start _is the starting time of treatment and *v*_1 _and *v*_2 _are the intensity parameters of treatment. A simulation of the effect of a proteasome inhibitor is given in Fig. 9 and Fig. 10, and corresponds to the untreated tumor simulations illustrated in Fig. 4 and Fig. 5. The tumor is extinguished, the osteoclast and osteoblast populations recover regular cycles, and the bone mass recovers to normal.

**Remark 1 ***A similar analysis can be made of other model parameters, as well as other treatment functions*.

The tumor is introduced at time 0, whereas treatment with a proteasome inhibitor is started at *t*_start _= 600. At this time, there is an increase in tumor mass and osteoclast number, and a decrease in osteoblast number, indicating the development of myeloma bone disease. Treatment with a proteasome inhibitor decreases tumor burden from the time of treatment, whereas there is a delay in the recovery of the bone mass. Analysis of the individual cell types (osteoclasts and osteoblasts) indicates a decrease in osteoclasts from time of treatment, but a delay in the expected increase in osteoblasts. This suggests that the reduction in osteoclast number is dependent on tumor burden, but is not sufficient to increase bone mass. The increase in bone mass appears to parallel the increase in osteoblast number. Taken together, this model suggests that proteasome inhibition has a dramatic effect to reduce tumor burden, and to prevent bone loss as well as increase bone formation to steady-state levels in multiple myeloma. These results are consistent with observations from both clinical studies and preclinical murine models, where tumor burden is decreased and bone volume or markers of bone formation are increased in response to bortezomib treatment [26,30-32]. Furthermore, by enabling analysis of tumor burden, bone volume and cell number over time, the results from this modeling provide important insights into the biology behind the clinical response to bortezomib which cannot be obtained from current *in vivo *studies.

### One-dimensional Bone Model without Tumor

**Figure 11:**
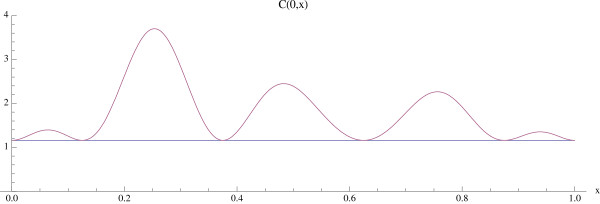
**The graph of the initial distribution *C*(0, *x*) for the bone model with an additional spatial dimension**. System of equations (14): Osteoclast numbers are initially elevated above the normal nontrivial steady state (*x*) ≡ 1.16 at multiple sites. The initial distribution *B*(0, *x*) = (*x*) ≡ 231.72 is taken as the normal constant nontrivial steady state.

The normal bone model in [22] is a discrete site model for single event remodeling and internally regulated cycles of remodeling. We reinterpreted bone mass as implicitly providing one spatial dimension, such as trabecular mass beneath a point on the bone surface. To incorporate additional dimensions of spatial variability, we develop a diffusion model in a second spatial domain Ω. For convenience here we take a one-dimensional region Ω = [0, 1]. We assume that both osteoclasts and osteoblasts are diffusing in Ω. The variables of the model are *C*(*t*, *x*) = density of osteoclasts and *B*(*t*, *x*) = density of osteoblasts at time *t *with respect to *x *∈ Ω. The equations are(25)

with boundary conditions(27)

and initial conditions *C*(0, *x*) = *C*_0_(*x*) and *B*(0, *x*) = *B*_0_(*x*) (see Fig. 11).

The nontrivial steady states  and  in the zero-dimensional case in Section are also steady states of the system (1) and viewed as constant functions  and  on Ω. Further, the change in bone mass *z*(*t*, *x*) as a function of *x *as well as *t *about a normalized value of 100 is given by the following equation: The value of 100 is arbitrary. Any other value, such as 1, could have also been used.(29)

with initial condition *z*(0, *x*) = *z*_0_(*x*), and boundary condition(30)

where σ_3 _is the diffusion coefficient for the bone mass, and *k*_1_(*x*) and *k*_2_(*x*) depend on *C*(0, *x*) and *B*(0, *x*). We remark that σ_3 _is typically small and represents stochasticity in the bone dynamics, not actual migration of bone stromal cells.

We give examples of the normal bone model for two cases of initial spatial inhomogeneity in the osteoclast population *C*(0, *x*). In both examples the nonspatial parameters are *α *_1 _= 3.0, *α *_2 _= 4.0, *β *_1 _= 0.2, *β *_2 _= .02, *g*_11 _= 1.1, *g*_22 _= 0.0, *g*_12 _= 1.0, *g*_21 _= -0.5 (as in Fig. 3, Part I), the spatial parameters are σ_1 _= .000001, σ_2 _= .000001, and the bone mass parameters are *σ *_3 _= .000001, *k*_1_(*x*) ≡ 0.45, *k*_2_(*x*) ≡ .0048, with *z*(0, *x*) ≡ 100.0. For these parameters the constant functions (*x*) ≡ 1.16, (*x*) ≡ 231.72 are steady-state solutions (see Fig. 3, Part I).

**Figure 12:**
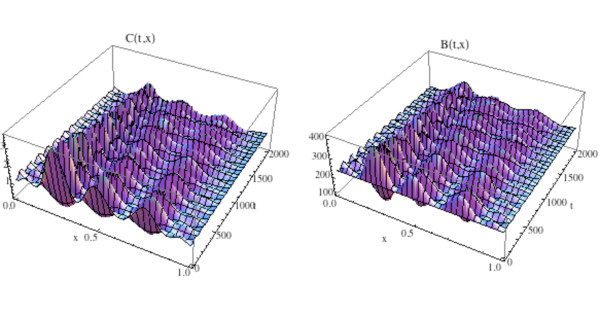
**Graphs of the solutions *C*(*t*, *x*) and *B*(*t*, *x*) of the bone model with an additional spatial dimension**. System of equations (14): We take *C*(0, *x*) as in Fig. 11 and *B*(0, *x*) = (*x*) ≡ 231.72. The solutions sustain regular spatial and temporal cycles characteristic of normal bone remodeling.

**Figure 13:**
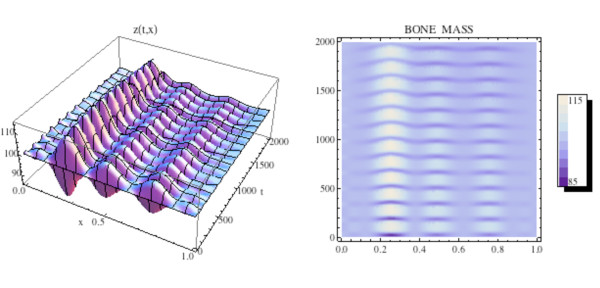
**Left side: Graph of the bone mass *z*(*t*, *x*) for the spatially dependent normal bone model with *C*(0, *x*) and *B*(0, *x*) as in Fig. 11 and *B*(0, *x*) = (*x*) ≡ 231.72. Right side: Density plot of the bone mass**. System of equations (14): The bone mass sustains regular spatial and temporal cycles uctuating about a normalized value of 100 dimensionless cell units.

We first simulate the normal bone model with the initial distribution of osteoclasts *C*(0, *x*) elevated above  = 1.16 near one site in Ω, as graphed in Fig. 12. The initial distribution of osteoblasts *B*(0, *x*) is taken as constant at  = 231.72. The density plots of the osteoclasts and osteoblasts, and the change in bone mass, are graphed in Fig. 13, where it is seen that the solutions sustain spatial and temporal cycles about these steady states.

### One-dimensional Bone Model with Tumor

For the spatial model with tumor we assume the tumor cells are also diffusing in Ω. The variables of the model are *C*(*t*, *x*) and *B*(*t*, *x*) as before, and *T*(*t*, *x*) = density of tumor cells at time *t *with respect to *x *∈ Ω.

The equations are(31)

with boundary conditions(34)

and initial conditions *C*(0, *x*) = *C*_0_(*x*), *B*(0, *x*) = *B*_0_(*x*), *T *(0, *x*) = *T*_0_(*x*). The bone mass equations for *z*(*t*, *x*) are as in (14e) and (14f). The diffusion coefficient for the tumor is σ_4_, which allows for spatial growth.

**Figure 14:**
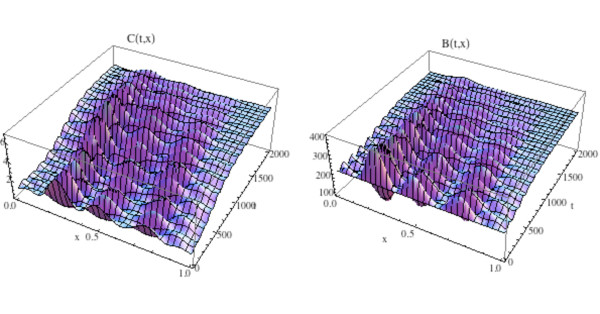
**Graphs of the solutions *C*(*t*, *x*) and *B*(*t*, *x*) of the spatially dependent bone model with tumor and with *C*(0, *x*) and *B*(0, *x*) as in Fig. 11**. System of equations (15): The solutions lose regular spatial and temporal cycles and converge to the nontrivial steady states  = 5.0,  = 316.0 (compare to Fig. 4 and Fig. 12).

**Figure 15:**
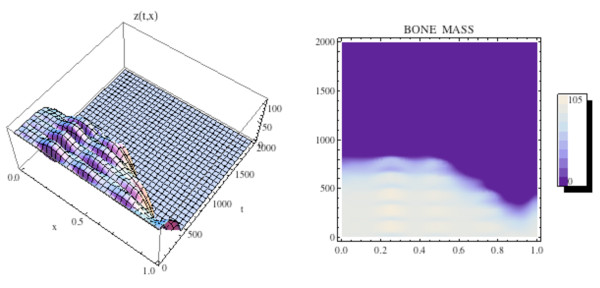
**The graph (left side) and density plot (right side) of the bone mass *z*(*t*, *x*) for the spatially dependent bone model with tumor**. System of equations (15): *C*(0, *x*) and *B*(0, *x*) are as in Fig. 11 (compare to Fig. 13).

**Figure 16:**
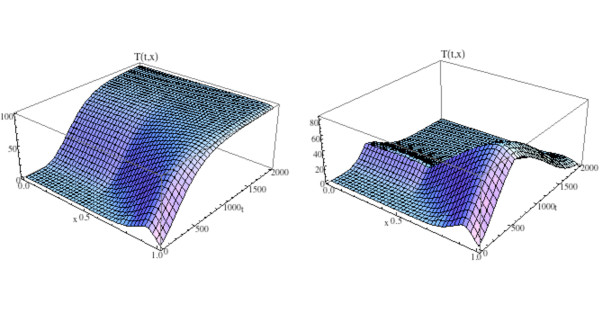
**Left side: Graph of the untreated tumor population *T*(*t*, *x*) for the spatially dependent bone model with tumor and with *C*(0, *x*) and *B*(0, *x*) as in Fig. 11**. System of equations (15): The tumor is initially small and located on the right side of Ω = [0, 1]. The tumor density *T*(*t*, *x*) converges to capacity *L*_*T *_for all *x *∈ Ω as time increases. Right side: Graph of the treated tumor population *T*(*t*, *x*). The tumor is extinguished as time increases.

We illustrate the tumor model with an additional space dimension by adding the tumor population to the simulation in Fig. 12 and Fig. 13. Tumor parameters are γ_*T *_= .004, *L*_*T *_= 100, *r*_11 _= .005, *r*_21 _= 0.0, *r*_12 _= 0.0, *r*_22 _= 0.2 (as in Fig. 3), the additional spatial parameters are σ_1 _= .000001, *σ *_2 _= .000001, σ_4 _= .000001, and the bone mass parameters are σ_3 _= .000001, *k*_1_(*x*) ≡ 0.45, *k*_2_(*x*) ≡ .0048, with *z*(0, *x*) ≡ 100.0. The zero-dimensional nontrivial steady state is  = 1.16,  = 231.72. In Fig. 14, 15, and the left side of Fig. 16 we simulate the model. The tumor is initially small and located on the right side of Ω = [0, 1]. Over time, as the tumor grows from the right side of Ω = [0, 1], the regular spatial and temporal cycles of the osteoclast and osteoblast populations are disrupted with these solutions ultimately approaching the zero-dimensional tumor model nontrivial steady state  = 5.0,  = 316.0, as in Fig. 4 (Fig. 14), the bone mass is depleted throughout Ω (Fig. 15), and the tumor grows to carrying capacity (Fig. 16, left side).

### One-dimensional Bone Model with Tumor and Treatment

The one-dimensional model with tumor and treatment is obtained by adding the time-dependent treatment functions *V*_1_(*t*) and *V*_2_(*t*) as in (13). The equations are(37)

with boundary conditions(40)

and initial conditions *C*(0, *x*) = *C*_0_(*x*), *B*(0, *x*) = *B*_0_(*x*), *T *(0, *x*) = *T*_0_(*x*). The bone mass equations for *z*(*t*, *x*) are again as in (14e) and (14f).

**Figure 17:**
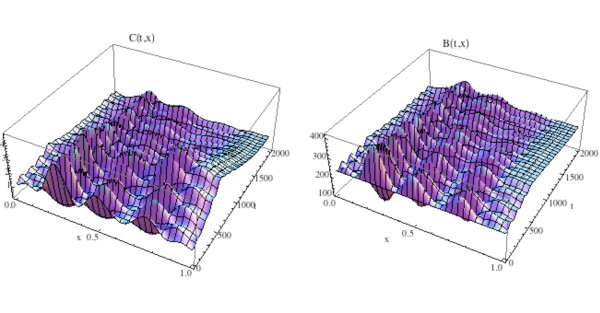
**Graphs of the solutions *C*(*t*, *x*) and *B*(*t*, *x*) for the spatially dependent bone model with tumor and treatment**. System of equations (16): The solutions recover regular spatial and temporal cycles after treatment begins at *t*_*start *_= 600 (compare to Fig. 12 and Fig. 14).

**Figure 18:**
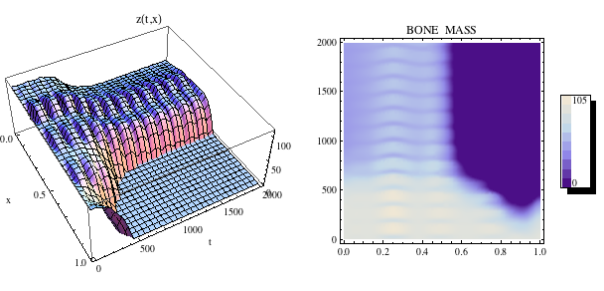
**The graph (left side) and density plot (right side) of the bone mass *z*(*t*, *x*) for the spatially dependent bone model with tumor and treatment (compare to Fig. 15)**. System of equations (16): Treatment stops the loss of bone mass from the advance of the tumor from the right side to the left side of the spatial region Ω = [0, 1] after initiation at *t*_*start *_= 600. Bone mass already lost is not recovered on the right side of Ω as treatment continues.

We illustrate the model in this section by adding treatment to the simulation in Fig. 14 and Fig. 15. All parameters and initial conditions are as in Fig. 11, 14, and 15. The treatment parameters are *v*_1 _= .0001 in (13b) and *v*_2 _= .006 in (13d) and *t*_*start *_= 600. This form of the treatment corresponds to drugs such as proteasome inhibitors, which promote osteoblast production and inhibit tumor growth. The graphs of osteoclast and osteoblast populations are given in Fig. 17, where both populations recover to regular cycles after the start of treatment at *t*_*start *_= 600. The bone mass is graphed in Fig. 18 and the tumor population is graphed on the right side of Fig. 16. The osteoclast and osteoblast populations recover normal cycling (Fig. 17), the bone mass recovers partially on the left side, but not on the right of Ω (Fig. 18), and the tumor is extinguished by the treatment (Fig. 16, right side).

## Discussion

We have presented a dynamic model of spatially heterogeneous bone remodeling that incorporates the interaction of osteoclasts (bone resorption) and osteoblasts (bone formation) subject to myeloma bone disease and its treatment. Our model is a system of nonlinear partial differential equations for osteoclast-osteoblast interactions driven by autocrine-paracrine signaling, which allows for interpretation of the corresponding spatial changes in bone mass and tumor growth. In the case of normal bone the model views remodeling as stable regular oscillations in a spatially heterogeneous microenvironment. In the case of myeloma, these regular cycles are destabilized with an increase in osteoclasts typical of this disease, and with corresponding destruction of bone mass and progressive tumor growth. Furthermore, additional features of myeloma bone disease are reproduced by this model, including the initial increase in osteoblast number, which is thought to reflect the attempt to maintain normal coupling of bone resorption to bone formation [15]. This is followed by a decrease in osteoblast oscillation below that of the non-tumor simulation, reflecting the decrease in osteoblasts which leads to reduced bone formation and subsequent bone loss in multiple myeloma. Of interest is the observation that osteoclast oscillations remain elevated above the non-tumor simulation at all times, despite an overall decrease reflecting the gradual bone loss. Again, this is highly representative of myeloma bone disease, which is associated with an increase in both osteoclast number and activity. We incorporated treatment into the model with a drug, such as a proteasome inhibitor, which promotes osteoblast production and inhibits tumor growth.

Proteasome inhibitors were initially identified by their dramatic effects to reduce myeloma tumor burden [28-30,32]. Subsequent studies suggested that this class of drugs may have additional effects in myeloma, due to their direct effects to promote osteoblast differentiation and subsequent bone formation [26-30]. In addition, it has been suggested that proteasome inhibitors may have effects on osteoclastic bone resorption [31,33,34]. In the current simulation, treatment with a proteasome inhibitor which had direct effects on myeloma cells and osteoblast formation was found to significantly reduce tumor burden and prevent myeloma bone disease, in agreement with reported *in vivo *preclinical studies. Of interest, effects on osteoclast number were also observed, suggesting that proteasome inhibitors may have indirect effects on osteoclasts. Osteoclasts and tumor burden were reduced from time of treatment, whereas there was a delay in the increase in osteoblasts and bone mass. This suggests firstly that the reduction in osteoclast number is dependent upon the tumor burden, and secondly that the increase in bone mass is a result of the increase in osteoblasts. Such insights into potential cellular mechanisms cannot easily be gained from *in vivo *experiments where cellular effects can typically only be assessed at endpoint, thus highlighting the value of this mathematical model. Furthermore, although the simulated proteasome inhibitor treatment was not able to completely restore bone mass, this is most likely a reflection of the rates chosen for the model and it is expected that bone mass would recover over a longer time period. This may suggest that the effects of proteasome inhibition to replace bone already lost in myeloma may take longer than the effects to reduce tumor burden, and have implications for duration of treatment.

The model we have developed is one-dimensional in space, and is thus idealized with respect to the geometry of the bone microenvironment. In future work we will develop a more realistic higher dimensional description of the bone microenvironment, and the dynamics of bone myeloma and its treatment in this spatial microenvironment. The major problem in the identification of therapeutic approaches for the treatment of multiple myeloma is the complex relationship between the multiple types of cells distributed throughout the bone marrow microenvironment, which is almost impossible to recreate *in vitro*. Therefore, in order to determine the effect of a drug, the optimal dose, treatment regimen or combination, and relative timing of the delivery of drug combinations, *in vivo *studies are required. Although the 5T murine model of myeloma is very effective and reliable for such preclinical studies, it is impossible to evaluate all drugs or combinations *in vivo*. Those that are selected for *in vivo *evaluation are often chosen based upon *in vitro *assays, which do not reflect the interactions within the bone marrow microenvironment.

## Conclusions

The frequencies of the osteoblast/osteoclast oscillations in the mouse are not yet known. Experimental determination of local trabecular density and osteoclast and osteoblast distributions in the 5T murine model requires histological examination of fixed sections of bone, precluding serial studies of the progression of multiple myeloma in an individual animal. The mathematical model should prove extremely useful in both minimizing the number of animals that have to be sacrificed to obtain statistically significant data regarding the time course of the disease, and in rationalizing inter-animal differences. Furthermore, the coupling between osteoblasts and osteoclasts, and bone remodeling cycle has yet to be recreated *in vitro*, either for normal bone or for diseased bone in the presence of myeloma cells. The current model accurately reflects myeloma bone disease and illustrates how treatment approaches may be investigated using such computational systems. Our ultimate goal is to quantify, with experimental support, the dynamics of bone remodeling in health and disease, and gain insight into the design and optimization of new therapeutic approaches for the treatment of myeloma bone disease.

## Methods

The mathematical analyses in this paper use accepted methodology from the theories of partial differential equations and dynamical systems. The computations use standard algorithms as incorporated into the software *Mathematica*.

## Competing Interests

The authors declare that they have no competing interests.

## Authors Contributions

All authors contributed to the development and interpretation of the mathematical model and the design of the figures. BPA and GFW developed the mathematical parts of the manuscript and conducted the simulations. All authors contributed to writing the manuscript, which was approved by all authors.

## Reviewers Comments

### Comments from Ariosto Silva

• I would suggest the authors to consider in their future additions to the model the effect of hypoxia in the bone formation equilibrium, mainly in the myelomatous bone marrow. Works have described the influence of hypoxia in the activity of osteoclasts [35] and pH in the activity of osteoblasts [36]. Considering that the bone marrow vascularization is not evenly distributed as in other tissues, gradients of oxygen concentration (and supposedly also of pHe) could induce niches where bone degradation is more favorable. This effect could also be exacerbated by the proliferation of MM cells which are known to have increased glucose consumption, as seen in PET scans.

*Response: We agree that hypoxia is an important aspect of the bone marrow microenvironment that will affect both bone cell activity and myeloma growth and survival, and as such, fully intend to incorporate hypoxia into future models*.

• In the conclusions, could you elaborate more on the reason of the increase in osteoblast activity in the early stages of MM?

*Response: During normal bone remodeling, osteoclastic and osteoblastic activity is tightly coupled, with an increase in osteoclast activity followed by an increase in osteoblast activity. In the early stages of myeloma, myeloma cells within the bone marrow stimulate an increase in osteoclast activity. Osteoblast activity is also increased in an attempt to maintain the normal coupling of bone remodeling. As the disease progresses, osteoblast formation and activity are inhibited, leading to dramatic bone loss*.

• Does the delay after the reduction of tumor burden and recovery of bone mass depend on the parameters of the model? Is it found in vivo and in clinical treatment as well? Could the delay in recovery of bone mass in patients be mapped into parameters from the model? Could this possibly be used as a prognosis for relapse or patient recovery?

*Response: The delay is dependent on model parameters. There is evidence to suggest that proteasome inhibition can increase bone formation in vivo, in addition to decrease tumor burden, however the time course of these events is unknown. This is an advantage of the mathematical model, that allows us to look in detail at all time points, whereas in vivo studies are limited to select time points which may not provide all information*.

• *"Those that are selected for in vivo evaluation are often chosen based upon in vitro assays, which do not reflect the interactions within the bone marrow microenvironment"*. Some in vitro models have been used to assess the effect of fibronectin and contact with stroma cells in chemoresistance in leukemia [37] and Multiple Myeloma [38]. It would be interesting indeed if data could be obtained from these in vitro models and ported into computational models like the one described in this work. *Response: There is substantial evidence to demonstrate that interactions between bone marrow stromal cells and myeloma cells are important for promoting tumor growth and survival, both alone and in response to chemotherapeutic agents. Future studies will incorporate bone marrow stromal cells into the mathematical models*.

• *"The mathematical model should prove extremely useful in both minimizing the number of animals that have to be sacrificed to obtain statistically significant data regarding the time course of the disease, and in rationalizing inter-animal differences"*. Depending on how you parameterize the model and which inputs you require from experiments, you may also created personalized models for each patient and even help on the prognosis of the disease or suggest more promising treatments for every patient.

*Response: The long-term goal of this modeling is to create a mathematical model which closely resembles human myeloma, and with which we can explore multiple treatment protocols and select the one that has the optimal outcome. This would represent a major step forward in individualized cancer therapy - quantitative optimization of a combination of drugs with a possibly complicated delivery schedule, all based upon a validated mathematical model*.

• Even though this work is an extension of a previous one already published [22] it would be useful to describe the values used for the parameters, if they were estimated or obtained from literature and if the authors have plans on how to obtain these form experiments for the future versions of the model.

*Response: The parameters for normal bone were taken from *[1,22]. *The parameters for myeloma bone with and without treatment were estimated. In future work the parameters will be identified using experimental data*.

### Comments from Mark P. Little

This is a generally well-written paper, describing a schematic model of bone remodelling in the presence or absence of myeloma. It was not immediately obvious what the relevance of this article is to the Biology Direct special issue, but perhaps this doesn't matter too much. Arguably more important are the specific modelling assumptions, in particular the power law ODEs and PDEs relating osteoclasts and osteoblasts. While these appear plausible, a little more biological justification of these functional forms could be usefully provided here (e.g., as is given in ref. [22]).

*Response: The theme of this Biology Direct special issue is Mathematics and the Evolution of Cancer. The manuscript outlines the development of a mathematical model of myeloma bone disease. Due to the interactions between myeloma cells and cells of the bone marrow microenvironment, the osteolytic bone disease associated with myeloma is inextricably linked with tumor progression, and therefore is relevant to this Biology Direct special issue. Power law nonlinearities have been used extensively in modelling biochemical kinetics in interacting systems. The parameters in the power law terms correspond to feedback and other regulatory mechanisms. In future work the power law parameters will be estimated using experimental data*.
